# Near complete response to Pembrolizumab in microsatellite-stable metastatic sebaceous carcinoma

**DOI:** 10.1186/s40425-018-0357-3

**Published:** 2018-06-19

**Authors:** Evidio Domingo-Musibay, Paari Murugan, Alessio Giubellino, Sandeep Sharma, Daniel Steinberger, Jianling Yuan, Matthew A. Hunt, Emil Lou, Jeffrey S. Miller

**Affiliations:** 1 Department of Medicine, Division of Hematology, Oncology and Transplantation, University of Minnesota, 420 Delaware Street SE, MMC480, Minneapolis, MN 55455 USA; 2 Department of Laboratory Medicine and Pathology, University of Minnesota, Minneapolis, MN 55455 USA; 3 Department of Radiology, University of Minnesota, Minneapolis, MN 55455 USA; 4 Department of Radiation Oncology, University of Minnesota, Minneapolis, MN 55455 USA; 5 Department of Neurosurgery, University of Minnesota, Minneapolis, MN 55455 USA; 6 Masonic Cancer Center, University of Minnesota, Minneapolis, MN 55455 USA

**Keywords:** Sebaceous carcinoma, Pembrolizumab, Immunotherapy, Anti-PD1, Skin cancer5

## Abstract

**Background:**

Sebaceous carcinoma is an aggressive adnexal skin tumor with a predilection for the eyelids and sebaceous glands of the head and neck.

**Case presentation:**

A 73 year-old man presented with confusion and was found to have widely disseminated sebaceous carcinoma with metastases to brain, lungs, liver, bowel, lymph nodes, and bone. Following initial treatment of the brain metastases with surgery he received post-operative radiosurgery. He then began systemic immunotherapy with pembrolizumab. After 6 months, he developed a near complete response to therapy by irRECIST and RECIST v.1.1. The response was associated with circulating CD8+ T cells with central memory (CM) and effector memory (EM) phenotype and mature CD16 + CD57+ NK cells. During treatment the patient developed adrenal insufficiency requiring high-dose systemic corticosteroids and later adrenal replacement therapy. After 12-months of follow-up he showed imaging evidence of progression in liver, mediastinum, and abdominal lymph nodes. Given persistent, strong PD-L1 expression he resumed pembrolizumab therapy and showed radiographic evidence of an ongoing response to therapy.

**Conclusions:**

This is the first report describing objective clinical and radiographic responses following immunotherapy for widely metastatic sebaceous carcinoma. The dramatic therapeutic response to pembrolizumab was associated with peripheral blood circulating memory T cells and mature Natural Killer cells after 6 months (24 weeks) of therapy. This report supports prospective clinical trials of anti-PD1 checkpoint blockade for metastatic sebaceous carcinoma.

**Electronic supplementary material:**

The online version of this article (10.1186/s40425-018-0357-3) contains supplementary material, which is available to authorized users.

## Background

Sebaceous carcinoma (SC) is an uncommon form of skin adnexal tumor, often arising from sebaceous glands of the eyelid [1], and head and neck [2]. Periocular primary tumors comprise more than half of cases [3], and sebaceous carcinoma (SC) is the third most common eyelid tumor, after basal cell and squamous cell carcinoma [1, 4]. The overall incidence is rising [4], but cases are estimated to be 1 to 2 per 1,000,000 in the US, based on a recent review of the Surveillance Epidemiology and End Results (SEER) database [5].

Optimal treatment of metastatic sebaceous malignancy has not been firmly established. To date, treatment approaches have been adapted from regimens used to treat head and neck cancers, with several retrospective series showing effectiveness of multi-agent cisplatin-based chemotherapy [6, 7]. While the sporadic form of SC is not generally associated with mutations in DNA mismatch repair genes, cases associated with Muir-Torre and microsatellite instability (MSI) are likely to respond to immunotherapy [8, 9]. Anti-PD1 checkpoint inhibitors are approved for malignant melanoma [10, 11] and merkel cell carcinoma, a polyomavirus associated skin adnexal tumor [12, 13]. To date, there have been no clinical trials or case reports describing successful use of immunotherapy for sebaceous carcinoma tumors.

## Case presentation

Herein, we describe the case of a 73 year-old man in good health, who developed widely disseminated sebaceous carcinoma including metastases to brain, visceral organs, lymph nodes, and bone.

**Fig. 1:**
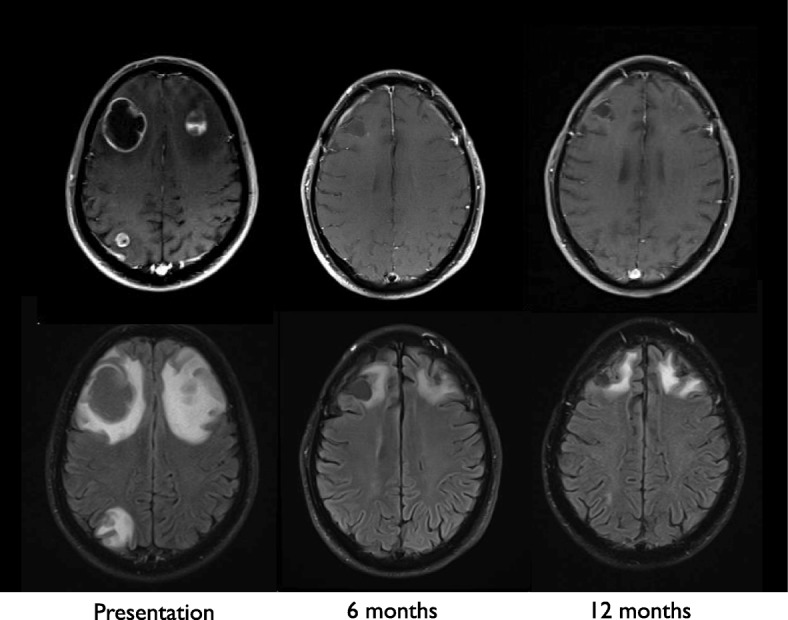
Magnetic Resonance Imaging (MRI) brain: T1-weighted images following intravenous gadolinium-based contrast (top panel) and axial FLAIR images without contrast (bottom panel). MRI brain images taken at initial presentation show two large frontal lobe enhancing lesions at the gray-white matter junction with significant surrounding edema and associated T2 FLAIR hyperintensity. Post-treatment changes remain evident at 6 and 12 month follow-up scans

He initially presented in late October 2016 for removal of a rapidly growing nodule in the anterior abdominal wall. Two days later he developed confusion, urinary incontinence and progressive aphasia. Emergent magnetic resonance imaging (MRI) of the brain showed 4 enhancing gray-white matter junctional lesions, the two largest measured 3.8 × 3.3 cm in the right frontal lobe and 2.3 × 2.5 cm in the left frontal lobe. Two smaller enhancing lesions in the right parietal lobe measured 8 mm and 4 mm in diameter. In November 2016, he underwent craniotomy and resection of bilateral frontal lobe tumors, and he made a full neurologic recovery and went on to receive post-operative gamma knife radiosurgery to the resection cavities and the small parietal brain lesions (Fig. 1).

**Fig. 2:**
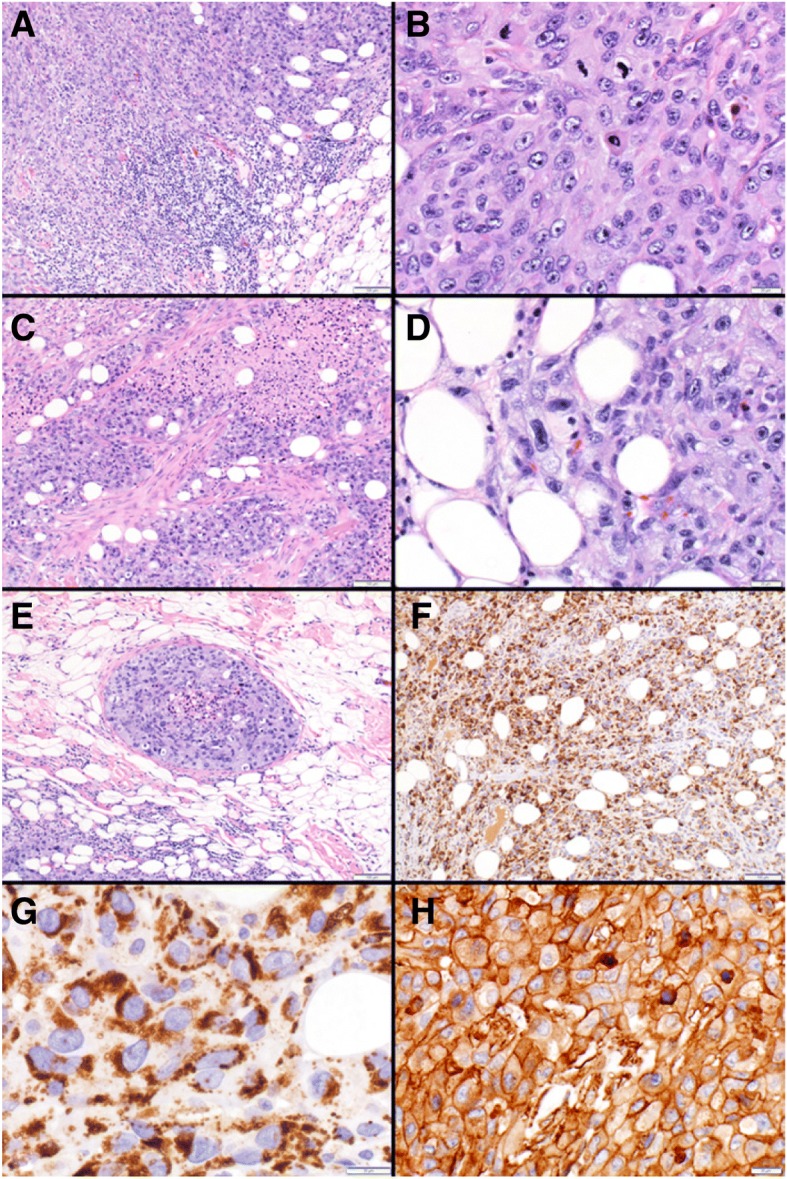
Sebaceous carcinoma: Sheets of malignant cells are shown invading subcutaneous adipose tissue along with tumor infiltrating lymphocytes (lower right) (**a**). Malignant epithelial cells with nuclear pleomorphism, coarse chromatin, prominent nucleoli and increased mitotic activity (**b**). Carcinoma with stromal desmoplasia and tumor necrosis (**c**). Intracytoplasmic vacuolations suggest sebaceous differentiation (**d**). Carcinoma exhibiting lymphovascular space invasion (**e**). Adipophilin immunohistochemistry demonstrating diffuse (**f**) and characteristic strong membranous staining of the intracytoplasmic lipid vacuoles (**g**). PD-L1 (22C3, pharm Dx) immunohistochemistry showing positive (high, tumor proportion score > 50%) expression in 100% of tumor cells (**h**)

His case was reviewed in melanoma tumor boards at the Masonic Cancer Clinic, University of Minnesota. Sections of tumor revealed sheets of epithelial cells with moderate eosinophilic cytoplasm and areas of tumor infiltrating lymphocytes (Fig. 2a). Cells exhibited nuclear pleomorphism and increased mitotic activity (Fig. 2b), desmoplastic stromal reaction and necrosis (Fig. 2c). Immunohistochemical staining was positive for cytokeratin AE1/AE3 and cytokeratin 7, and negative for S100, HMB45, Melan-A, CD45, calretinin, ERG, p40, TTF1, CDX2, and GATA3. The immunoprofile ruled out melanoma, mesothelioma, lymphoma, sarcoma with epithelioid features, and most visceral carcinomas. Microscopic examination revealed intracytoplasmic lipid vesicles (Fig. 2d), confirmed by diffuse membranous reactivity for adipophilin [14, 15] (Fig. 2f and g). The findings supported a histopathologic diagnosis of sebaceous carcinoma. Importantly, additional tumor testing confirmed high expression of PD-L1 in 100% of tumor cells (Fig. 2h). Commercial genomic testing using next-generation sequencing (Foundation Medicine, Massachusetts, USA) confirmed the tumor was microsatellite stable and carried a mutational burden of 17 mutations/Mb. Table 1 also shows various somatic mutations in genes for regulatory transcription factors, DNA repair proteins, growth factor receptors, and targetable MAPK signaling proteins. Several of the affected genes have also been described in cases of sebaceous carcinoma reported in the COSMIC (cancer.sanger.ac.uk) database [16].

**Table 1:** Tumor genomic next generation sequencing (NGS) study reveals mutations in several genes, including somatic mutations in genes mutated in the sebaceous carcinoma COSMIC dataset (in bold)

Gene	Alteration	Subcellular localization	Pathway
LRP1B	G3156C, Q1125*	Plasma membrane	Receptor mediated endocytosis
CCND1	S41L	Nucleus; cytosol	Cyclin D1, cell cycle
**TET2**	P174H	Nucleoplasm	DNA demethylation
**TP53**	rearrangement, del exon 10-11	Nucleoplasm	DNA repair
FANCA	A816V, R685S	Nucleus	DNA repair
FGF6	A63T	Extracellular	Growth factor
MYST3	Q1681_Q1684del	Nucleolus; cytosol	Histone acetyltransferase (HAT)
**KRAS**	G12C	Cytosol	MAPK signalling
RBM10	F173fs*7	Nucleus	mRNA splicing
**MET**	amplification	Plasma membrane	Receptor tyrosine kinase
**FGFR3**	I539del	Endoplasmic reticulum	Receptor tyrosine kinase
FLT4	K520E, R658W	Nucleus; plasma membrane	Receptor tyrosine kinase
ROS1	N692H	Vesicles	Receptor tyrosine kinase
TERT	-124C>T	Nucleoplasm	Telomerase
WT1	R471S	Nucleoplasm	WT1 Transcription Factor
MYC	L435F	Nucleoplasm	MYC Transcription Factor
ZNF703	G406R	Nucleus	Transcriptional co-repressor
c11orf30	C1211S	Nucleoplasm	Trascriptional Repressor
FLCN	R320Q	Nucleus; cytosol	Tumor suppressor
**TNFAIP3**	R706Q	Cytosol	Ubiquitination

*denotes mutations causing a premature stop codon

fs denotes the presence of a frameshift mutation

**Fig. 3:**
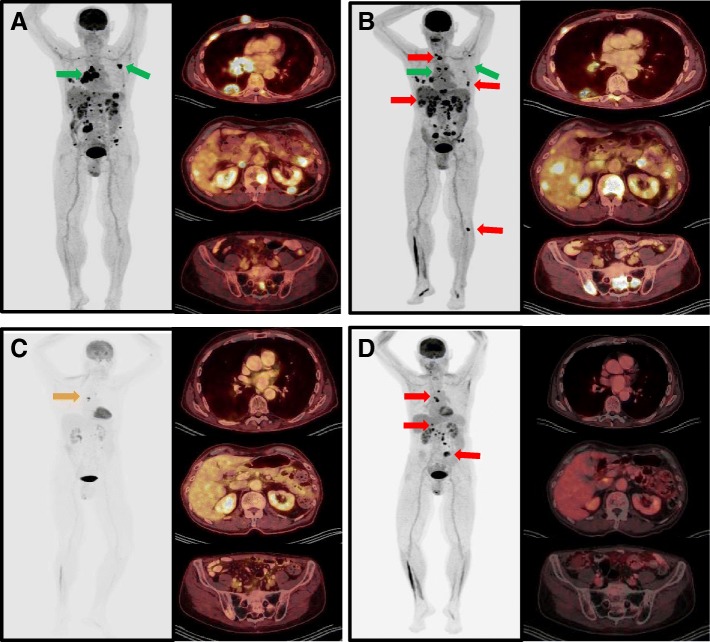
Positron emission tomography (PET) maximum intensity projection (MIP) images and axial images from the chest, abdomen, and pelvis demonstrating the radiographic response to pembrolizumab therapy. **a** Baseline imaging shows extensive metastatic disease burden, and green arrows highlight lesions first to improve in lung, mediastinum, and axillary lymph nodes. **b** Imaging after 3 months (4 cycles) of pembrolizumab are consistent with mixed response and pseudoprogression, with several new hypermetabolic foci (red arrows) in soft tissue, liver and bone. **c** Imaging studies at 6 months shows a near complete response to 7 cycles of pembrolizumab, with regression of multiple hypermetabolic metastatic foci, and few small remaining foci of residual hypermetabolism in the chest and abdomen. **d** Imaging at 12 months shows persistent FDG avidity in mediastinal and abdominal lymph nodes and new FDG avid lesions in liver and small bowel (red arrows)

Initial staging positron emission tomography-computed tomography (PET/CT) revealed evidence of widely disseminated disease involving lung and liver, muscle, bone, and multi-compartment bulky lymphadenopathy in chest and abdomen (Fig. 3a). Standard chemotherapy approaches using platinum-based chemotherapy were reviewed. However, the patient and family strongly favored a less toxic therapy, considering advanced age and quality of life concerns. Given the strong rationale for use of checkpoint inhibitors in several other tumor types, moderately high tumor mutational burden (17 muts/Mb), and strong PD-L1 expression the patient opted for anti-PD1 immunotherapy. He initiated off-label treatment with pembrolizumab (2 mg/kg, every 3 weeks) in December 2016.

**Fig. 4:**
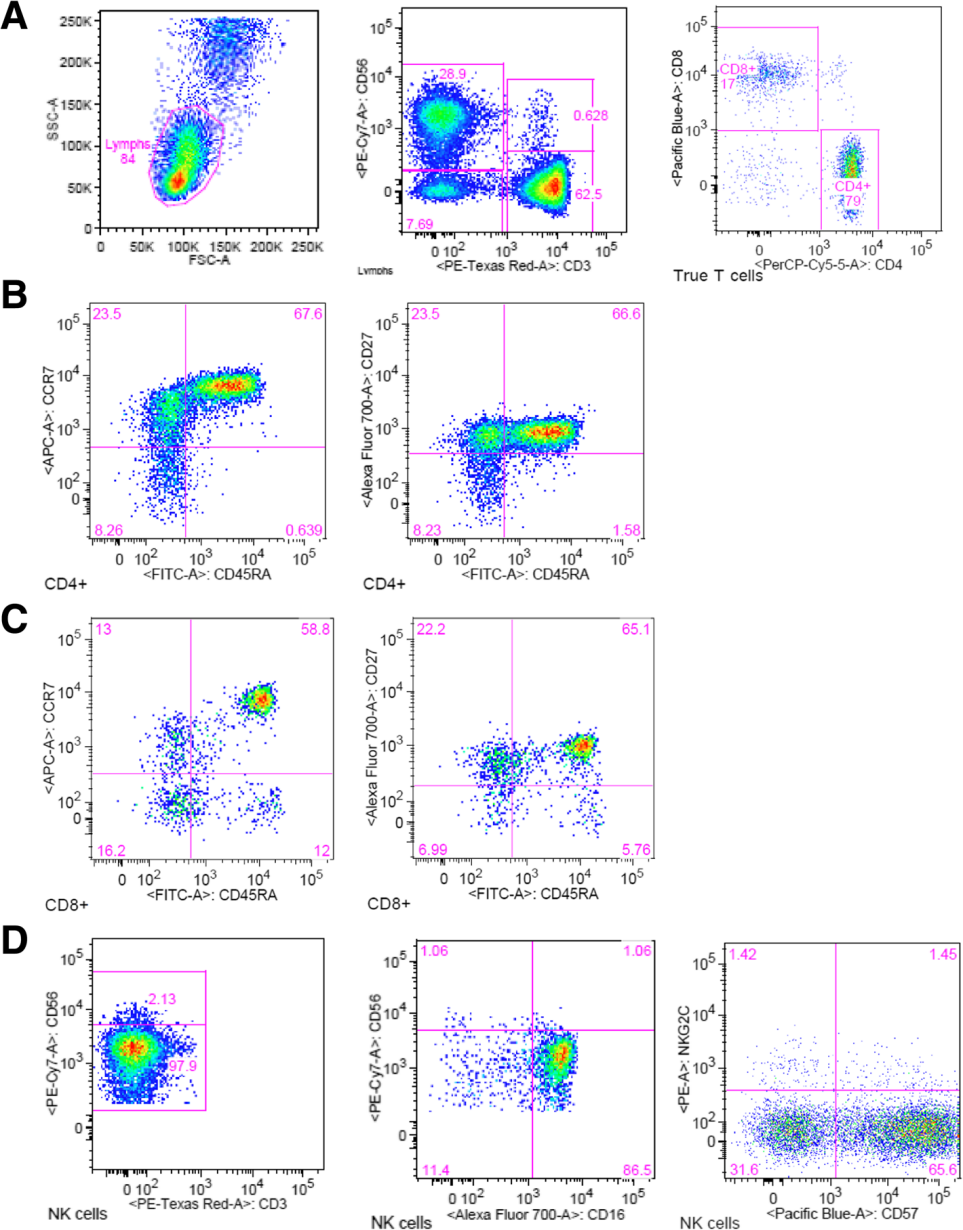
Peripheral blood mononuclear cell (PBMC) phenotyping at 6-month (24 week) follow-up visit. **a** Dot plots show lymphocyte gating and the relative frequency of CD3-CD56+ NK cells and CD3+ T cells. **b** Dot plots show relative frequency of CD4 + CD45RA- memory T cells expressing CCR7 and CD27, and CD127^lo^ CD25+ regulatory T cells. **c** Dot plots of CD3 + CD8+ gated cells show that CD45RA-CD8+ T cells stain for memory markers CCR7 and CD27, and there are smaller populations of CD45RA + CCR7- and CD45RA + CD27- effector T cells. **d** NK cells are mature (CD56 dim), terminally differentiated (CD57+) and express the Fc-gamma receptor, CD16. Numbers inside dot plots indicate the percentage of cells for the markers analyzed

Follow up PET/ CT scan 3 months after the initiation of anti-PD1 therapy revealed remarkable improvement in lymph nodes, lung, and soft tissue, however, there were multiple new and enlarging hepatic and osseous metastases initially worrisome for progression(Fig. 3b). After multidisciplinary review, the findings were felt consistent with pseudo-progression and immunotherapy was continued. Restaging PET/ CT obtained after 6 months of treatment showed further significant improvements in all previously noted lesions (Fig. 3c), with residual FDG activity seen in small mediastinal and abdominal lymph nodes. To further characterize the patient’s innate and adaptive immune status at the time of his near complete response, a flow cytometry study of peripheral blood was performed. Lymphocyte subset analysis showed evidence of circulating CD45RA-CD27+ central memory (CM) and effector memory (EM) T cells, and a population of mature CD16 + CD57+ NK cells (Fig. 4).

He continued on pembrolizumab, however, after 10 months of therapy he developed severe fatigue and orthostatic hypotension requiring hospitalization. Laboratory testing showed him to have secondary adrenal insufficiency with low (< 0.7 mcg/dL) serum cortisol and low (< 11 pg/mL) ACTH levels. He began 1 mg/kg prednisone with a prolonged steroid taper, and during high dose steroid therapy pembrolizumab dosing was interrupted. In December 2017, with 12 months of follow-up, restaging PET/CT was obtained and showed new FDG avid mediastinal and abdominal lymph nodes and a new hepatic and small bowel lesion (Fig. 3d). Biopsy of the liver lesion in January 2018 confirmed recurrent metastatic sebaceous carcinoma, and repeat immunohistochemical staining showed tumor cells continued to express high levels of PD-L1 (not shown). After discussion, the patient elected to restart immunotherapy, and he was continued on maintenance adrenal replacement therapy with hydrocortisone (10 mg/5 mg). Recent restaging studies demonstrate growth of the mediastinal metastases and a reduction in the size of the hepatic and small bowel metastases, suggestive of pseudoprogression and a durable ongoing response to pembrolizumab (Additional file 1).

## Discussion and conclusions

This is the first report of immune checkpoint inhibition in a patient with widely disseminated sebaceous carcinoma and brain metastases. Our patient was initially treated with brain-directed therapy and he recovered normal neurologic function. Systemic immunotherapy with anti-PD1 blockade led to a dramatic near complete response following 6 months of therapy. Initial tumor samples demonstrated strong and uniform PD-L1 expression on 100% of tumor cells (Fig. 1h), and the biopsy confirmed hepatic recurrence similarly expressed high levels of PD-L1. The ongoing response to pembrolizumab supports PD-L1 expression may be the most important biomarker predicting benefit from anti-PD1 therapy in sebaceous carcinoma. PD-L1 expression has been associated with response to pembrolizumab in advanced cutaneous melanoma [17], basal cell carcinoma [18], merkel cell carcinoma [19], and other tumors [20–22].

Several therapeutic targets derived from next generation sequencing were identified that could inform personalized and combinatorial treatment strategies. For example, it is worthwhile to note amplification of the MET gene, whose involvement in tumor progression and metastasis in several cancers has been well documented [23–28]. This finding may be relevant in terms of potential combination therapy, with the ultimate goal of accomplishing a durable, synergistic therapeutic response. The combination of pembrolizumab and the c-Met inhibitor crizotinib is currently in phase 2 testing for Alk-positive non-small cell lung cancer (NCT02511184). The alterations in FGFR and FLT4 are similarly attractive targets for combination therapy in view of the antiangiogenic effects of their inhibition [29, 30].

The immunologic phenotyping study performed at 6 months (24 weeks) of therapy demonstrated that our patient’s clinical response was associated with circulating CD8+ cytotoxic T lymphocytes in peripheral blood. The cytotoxic CD8 T cells had both central memory and effector memory phenotypes (Fig. 4c), as has been reported following vaccination against yellow fever and small pox [31]. Ribas and colleagues have also previously shown that tumor infiltrating CD8 memory T cells are associated with the response to anti-PD-1 therapy [32]. In anti-PD1 responders, memory CD8 T cells increased within the tumor microenvironment compared with non-responders after an average of 10 weeks; range 15-230 days [32]. While we did not specifically assess tumor infiltrating lymphocytes, our patient’s clinical response suggests cytotoxic memory T cells may persist in the peripheral blood circulation. We also saw smaller populations of circulating effector T cells, similar to the report by Kamphorst and colleagues in non-small cell lung cancer patients responding to anti-PD1 immunotherapy [33]. Peripheral blood collected within 4 to 24 weeks of treatment in that study, showed increases in Ki-67 + CD8+ T cells lacking CD45RA and CCR7, consistent with T effector cell phenotype. Importantly, Ki-67 + CD8+ T cells also expressed high levels of PD-1 and CTLA-4, and CD28 and CD27 costimulatory molecules [33].

We hypothesize that chronic antigen stimulation by sebaceous carcinoma can stimulate adaptive immunity and T cell exhaustion, enabling the use of anti-PD1 checkpoint inhibition strategies. These preliminary results appear promising for this tumor histology, however, prospective clinical trials are needed. Early phase testing in the cooperative groups and clinical trial networks may enable more rapid translation of these findings to the clinic and support the tumor immunology research and centralized testing of clinical trial samples. Further detailed tumor antigen discovery and immune profiling during treatment of additional patients would allow for a clearer understanding of the adaptive immune response against both MSS and MSI-high sebaceous carcinoma tumors.

In conclusion, the ongoing, durable response to checkpoint inhibition described in this report supports clinical testing of anti-PD1 checkpoint inhibitors in MSS and MSI-high sebaceous carcinoma. Prospective, open-label clinical testing is warranted to further define the role of front-line immunotherapy for the treatment of advanced, metastatic sebaceous carcinoma.

## Additional file

Additional file 1 : **Figure S1.** Coronal and axial contrast enhanced CT-images were obtained at 13 months and shows mediastinal and liver recurrence. Repeat imaging at 15 months shows increased size of the mediastinal lesion (top row), and decreased size of the hepatic lesion (bottom row), suggesting mixed immune related responses and pseudoprogression. (DOCX 600 kb)
