# Supplementary material for: Near complete response to Pembrolizumab in microsatellite-stable metastatic sebaceous carcinoma
Source: J Immunother Cancer. 2018 Jun 19;6:58. doi: 10.1186/s40425-018-0357-3 (PMC6006706; doi:10.1186/s40425-018-0357-3)
Supplement: Supplementary file 1 — : Figure S1. Coronal and axial contrast enhanced CT-images were obtained at 13 months and shows mediastinal and liver recurrence. Repeat imaging at 15 months shows increased size of the mediastinal lesion (top row), and decreased size of the hepatic lesion (bottom row), suggesting mixed immune related responses and pseudoprogression. (DOCX 600 kb) [file 40425_2018_357_MOESM1_ESM.docx]

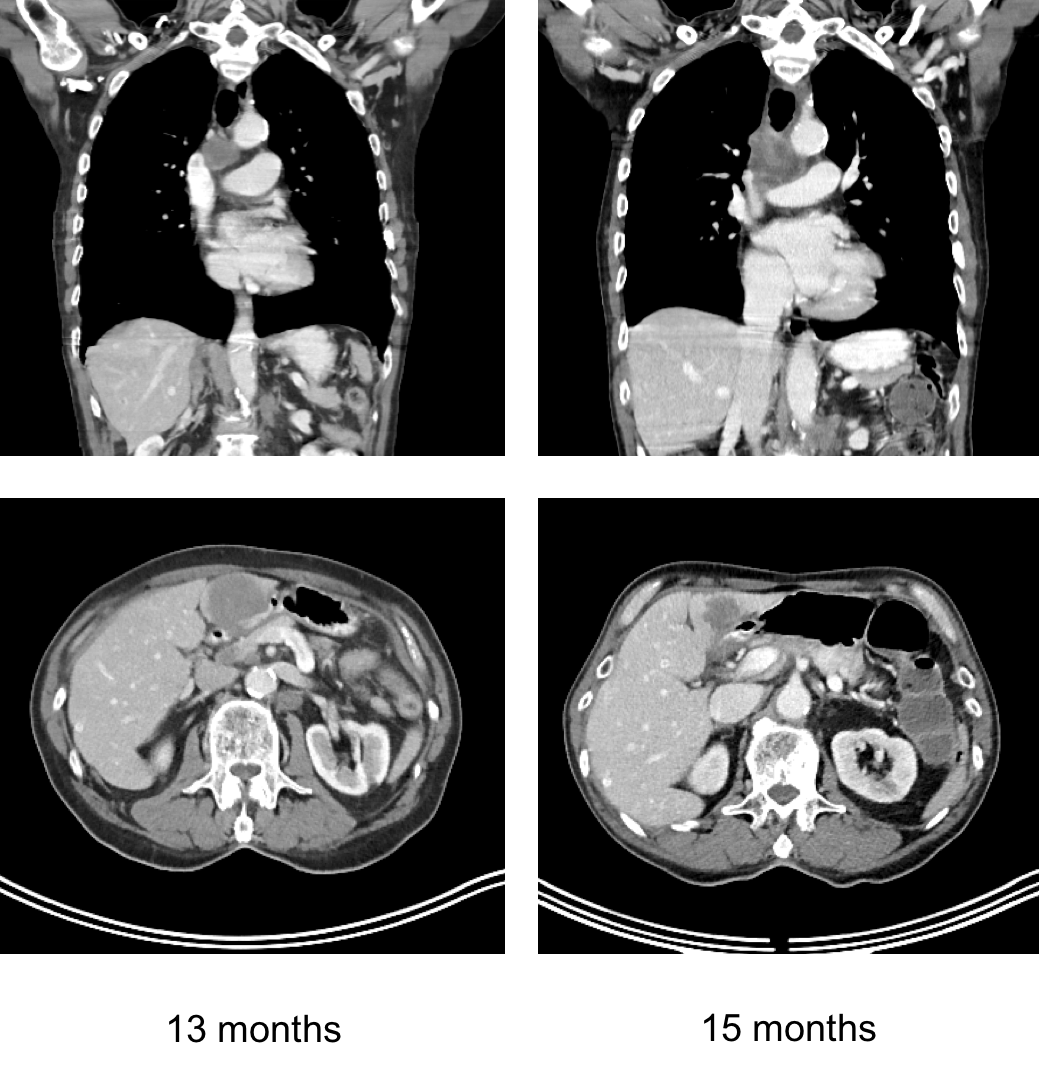


**Figure S1**: Coronal and axial contrast enhanced CT-images were obtained at 13 months and shows mediastinal and liver recurrence. Repeat imaging at 15 months shows increased size of the mediastinal lesion (top row), and decreased size of the hepatic lesion (bottom row), suggesting mixed immune related responses and pseudoprogression.
